# Supplementary material for: Iron induces two distinct Ca2+ signalling cascades in astrocytes
Source: Commun Biol. 2021 May 5;4:525. doi: 10.1038/s42003-021-02060-x (PMC8100120; doi:10.1038/s42003-021-02060-x)
Supplement: Supplementary file 2 — Supplementary Information [file 42003_2021_2060_MOESM2_ESM.pdf]

Iron induces two distinct Ca^{2+} signalling cascades in astrocytes

Wenzheng Guan, Maosheng Xia, Ming Ji, Beina Chen, Shuai Li, Manman Zhang, Shanshan Liang, Binjie Chen, Wenliang Gong, Chengyi Dong, Gehua Wen, Xiaoni Zhan, Dianjun Zhang, Xinyu Li, Yuefei Zhou, Dawei Guan, Alexei Verkhratsky and Baoman Li

Supplementary figures: Supplementary figure 1-4.

Supplementary Figure 1

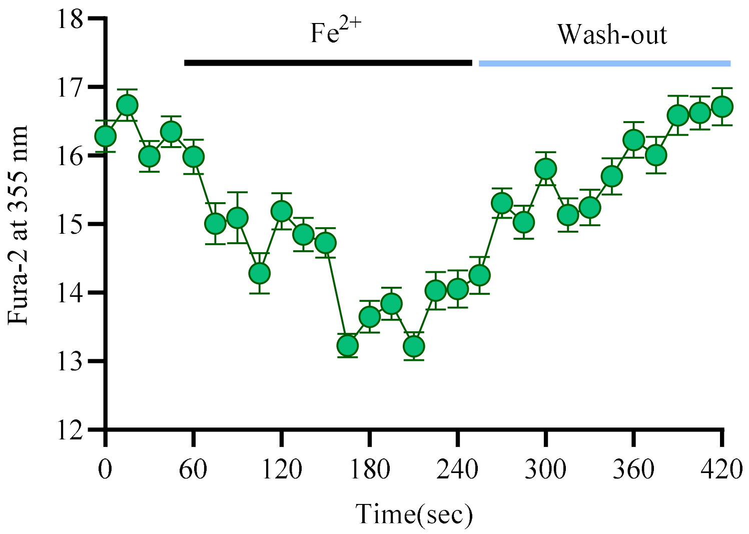

Supplementary Figure 1. Kinetics of Fe^{2+} entry into cultured astrocytes as assessed by quenching of quenching of the fura-2 signal excited at 355 nm. The value of fura-2 at 355 nm induced by Fe^{2+} was recorded. Every data point represents mean ± SD, n=6.

Supplementary Figure 2

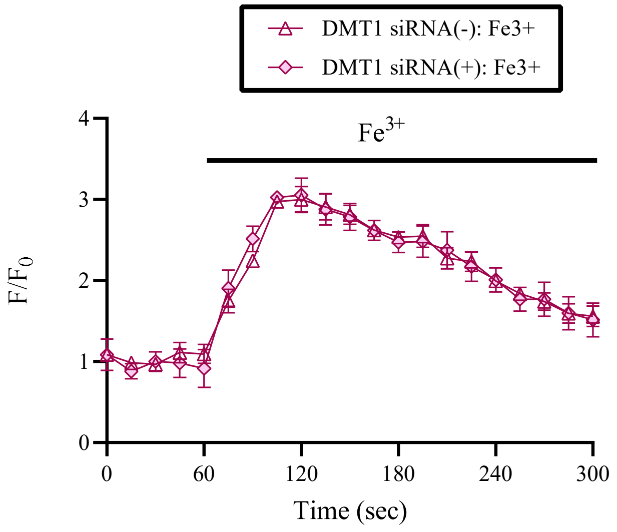

Supplementary Figure 2. Silencing of DMT1 expression by RNA interference does not affect Fe^{3+} -induced Ca^{2+} signalling in cultured astrocytes.

Representative $[Ca^{2+}]_i$ traces in response to application of 100 µM by Fe^{3+} after treatment with DMT1 siRNA negative control (-) or positive duplex chains (+) for 3 days. Every data point represents mean ± SD, n = 10, the experiment was repeated in 10 different cultures.

Supplementary Figure 3

Figure 2d

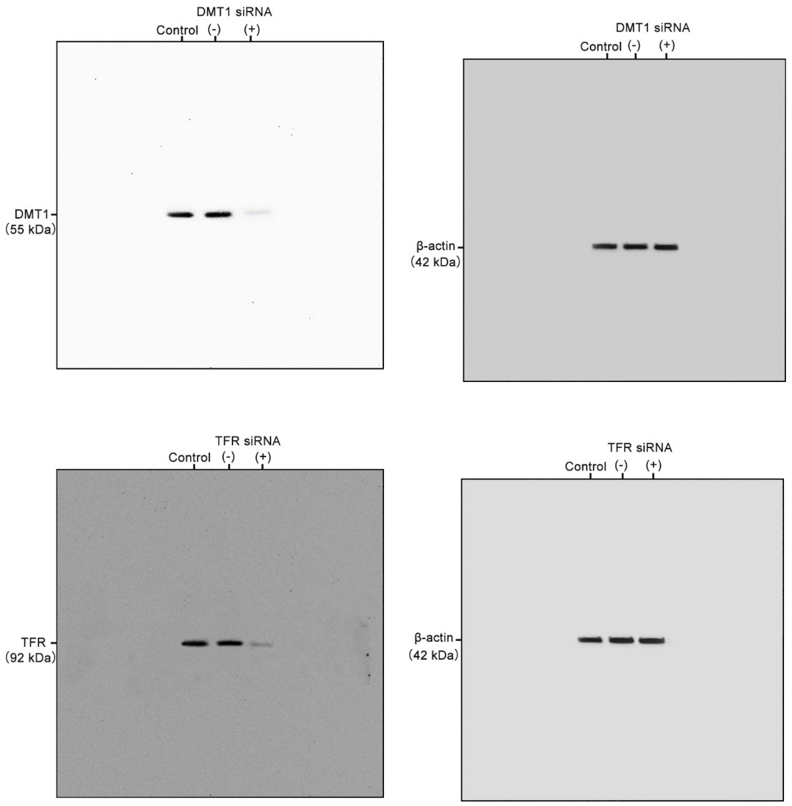

Figure 2g

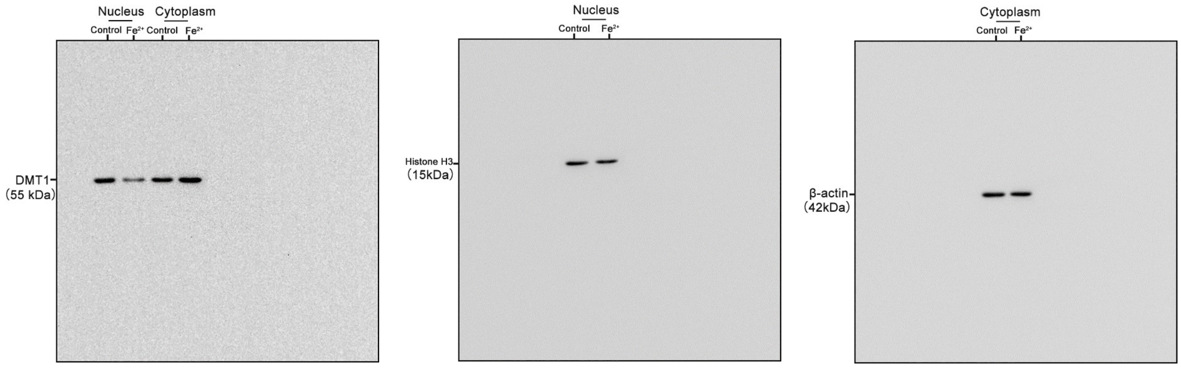

Supplementary Figure 3. Uncropped gel images of figure 2.

Supplementary Figure 4

Figure 4e

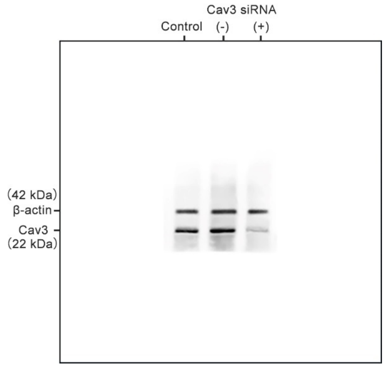

Figure 5a

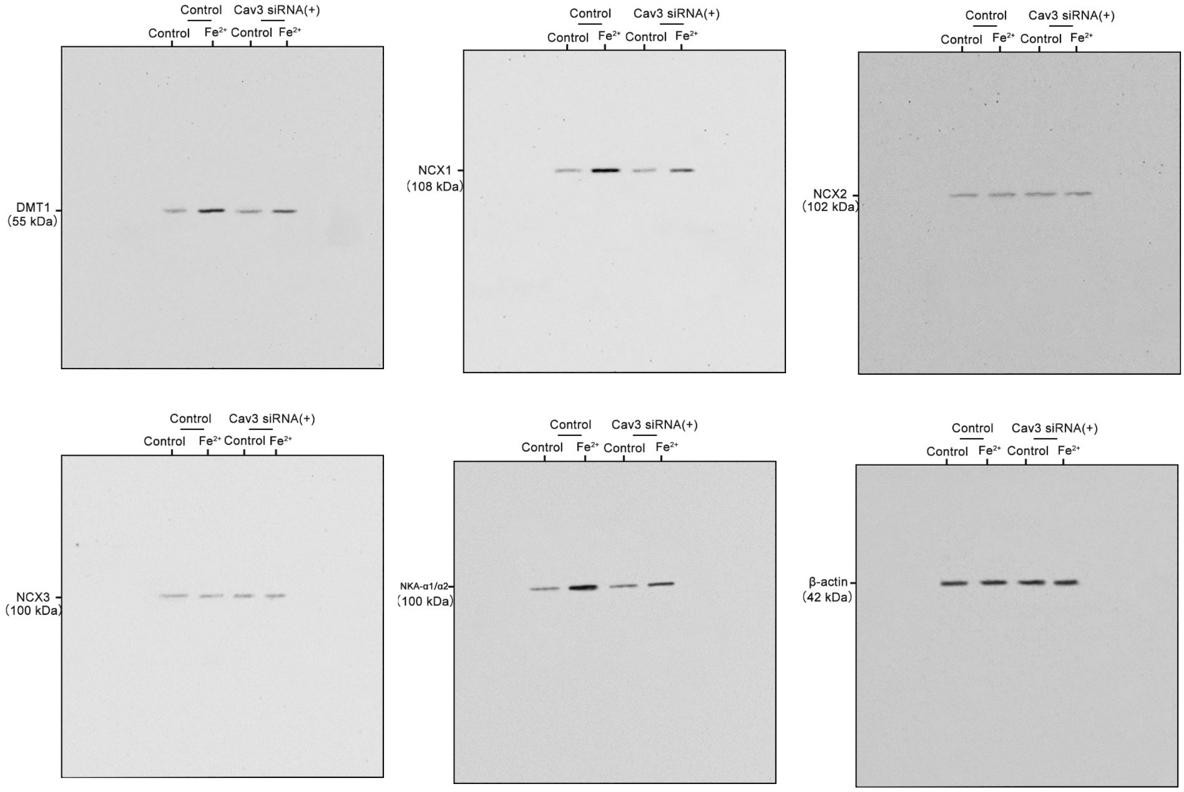

Figure 6b

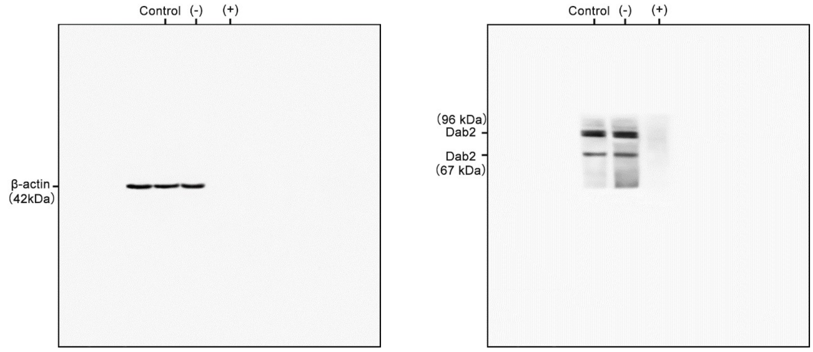

Supplementary Figure 4. Uncropped gel images (Fig.4, Fig. 5 and Fig. 6).
