# Supplementary material for: Iron induces two distinct Ca2+ signalling cascades in astrocytes
Source: Commun Biol. 2021 May 5;4:525. doi: 10.1038/s42003-021-02060-x (PMC8100120; doi:10.1038/s42003-021-02060-x)
Supplement: Supplementary file 5 — Reporting Summary [file 42003_2021_2060_MOESM5_ESM.pdf]

nature research

Corresponding author(s): Baoman Li

Last updated by author(s): Feb 17, 2021

Reporting Summary

Nature Research wishes to improve the reproducibility of the work that we publish. This form provides structure for consistency and transparency in reporting. For further information on Nature Research policies, see our Editorial Policies and the Editorial Policy Checklist.

Statistics

For all statistical analyses, confirm that the following items are present in the figure legend, table legend, main text, or Methods section.

n/a	Confirmed	
☐	☒	The exact sample size (n) for each experimental group/condition, given as a discrete number and unit of measurement
☐	☒	A statement on whether measurements were taken from distinct samples or whether the same sample was measured repeatedly
☐	☒	The statistical test(s) used AND whether they are one- or two-sided<br>*Only common tests should be described solely by name; describe more complex techniques in the Methods section.*
☐	☒	A description of all covariates tested
☐	☒	A description of any assumptions or corrections, such as tests of normality and adjustment for multiple comparisons
☐	☒	A full description of the statistical parameters including central tendency (e.g. means) or other basic estimates (e.g. regression coefficient) AND variation (e.g. standard deviation) or associated estimates of uncertainty (e.g. confidence intervals)
☐	☒	For null hypothesis testing, the test statistic (e.g. F, t, r) with confidence intervals, effect sizes, degrees of freedom and P value noted<br>*Give P values as exact values whenever suitable.*
☒	☐	For Bayesian analysis, information on the choice of priors and Markov chain Monte Carlo settings
☐	☒	For hierarchical and complex designs, identification of the appropriate level for tests and full reporting of outcomes
☐	☒	Estimates of effect sizes (e.g. Cohen's d, Pearson's r), indicating how they were calculated

Our web collection on statistics for biologists contains articles on many of the points above.

Software and code

Policy information about availability of computer code

Data collection	GraphPad Prism 5 software. One-way analysis of variance (ANOVA) followed by a Fisher's least significant difference (LSD) test for unequal replications using GraphPad Prism 5 software.
Data analysis	All data were expressed as mean and standard deviation (SD) and were analyzed using Student's t test or one-way analysis of variance with a value of <0.05 considered to be statistically significance.

For manuscripts utilizing custom algorithms or software that are central to the research but not yet described in published literature, software must be made available to editors and reviewers. We strongly encourage code deposition in a community repository (e.g. GitHub). See the Nature Research guidelines for submitting code & software for further information.

Data

Policy information about availability of data

All manuscripts must include a data availability statement. This statement should provide the following information, where applicable:

- Accession codes, unique identifiers, or web links for publicly available datasets
- A list of figures that have associated raw data
- A description of any restrictions on data availability

The data that support the findings of this study are available from the corresponding author upon reasonable request.

Field-specific reporting

Please select the one below that is the best fit for your research. If you are not sure, read the appropriate sections before making your selection.

- [x] Life sciences
- [ ] Behavioural & social sciences
- [ ] Ecological, evolutionary & environmental sciences

For a reference copy of the document with all sections, see nature.com/documents/nr-reporting-summary-flat.pdf

Life sciences study design

All studies must disclose on these points even when the disclosure is negative.

Sample size	The number of samples in RNA interfering are 6. In the other experiments (Ca2+ and Na+ signalling measurement, two photon and ELISA), the number of samples are 10.
Data exclusions	No data are excluded.
Replication	We confirm that the measurements in this study were successful replicated.
Randomization	The experimental animals or the cultured cells were randomly assigned to different experimental groups with a random number table.
Blinding	Western blotting, ELISA and the immunofluorescence tensity of Ca2+ or Na+ were performed by an investigator blinded to the experimental conditions.

Reporting for specific materials, systems and methods

We require information from authors about some types of materials, experimental systems and methods used in many studies. Here, indicate whether each material, system or method listed is relevant to your study. If you are not sure if a list item applies to your research, read the appropriate section before selecting a response.

Materials & experimental systems

n/a	Involved in the study	
☐	☒	Antibodies
☒	☐	Eukaryotic cell lines
☒	☐	Palaeontology and archaeology
☐	☒	Animals and other organisms
☒	☐	Human research participants
☒	☐	Clinical data
☒	☐	Dual use research of concern

Methods

n/a	Involved in the study	
☒	☐	ChIP-seq
☒	☐	Flow cytometry
☒	☐	MRI-based neuroimaging

Antibodies

Antibodies used	TFR antibody, β-actin antibody, GFAP antibody and DMT1 antibody were from Thermo Fisher Scientific (Waltham, MA USA); NCX1-3, Cav-3 and Dab2, NCX2 antibody, Na+/K+-ATPase alpha1/2 antibody and the secondary antibodies were bought from Santa Cruz Biotechnology (Santa Cruz, CA, USA); NCX1 antibody, NCX3 antibody were from Abcam (Cambridge, MA, USA); Secondary antibody staining with donkey anti-mouse or anti-rabbit Cy-2/3 were from Jackson Immuno-Research (West Grove, PA, USA). Primary antibody of histone H3 was purchased from EarthOx (Millbrae, CA, USA).
Validation	All primary antibodies all have mouse species reactivity and could be applied in western-blotting, immuno-fluorescence or co-immunoprecipitation.

Animals and other organisms

Policy information about studies involving animals; ARRIVE guidelines recommended for reporting animal research

Laboratory animals	The C57BL/6 mice, FVB/N-Tg(GFAP-eGFP)14Mes/J and B6.Cg-Tg(Thy1-YFP)HJrs/J transgenic mice were all purchased from the Jackson Laboratory (Bar Harbor, ME, USA). For the primary culture, the mice are newborn. For collecting cells, the transgenic mice were 12-16 weeks old. In vivo, two-photon, adult FVB/N-Tg(GFAP-eGFP)14Mes/J transgenic mice (10 to 12 weeks old) were anesthetized with ketamine (80 mg/kg, i.p.) and xylazine (10 mg/kg, i.p.). Body temperature was monitored using a rectal probe, and the mice were maintained at 37°C by a heating blanket. Half male and half female were used in this study.
Wild animals	The study does not involved wild animals.

Field-collected samples	The animals were raised in standard housing conditions (22 ± 1℃; light/dark cycle of 12/12h), with water and food available ad libitum.
Ethics oversight	All experiments were performed in accordance with the US National Institutes of Health Guide for the Care and Use of Laboratory Animals (NIH Publication No. 8023) and its 1978 revision, and all experimental protocols were approved by the Institutional Animal Care and Use Committee of China Medical University, No. [2019]059.

Note that full information on the approval of the study protocol must also be provided in the manuscript.
